# Supplementary material for: Target-dependent RNA polymerase as universal platform for gene expression control in response to intracellular molecules
Source: Nat Commun. 2023 Nov 17;14:7256. doi: 10.1038/s41467-023-42802-5 (PMC10656481; doi:10.1038/s41467-023-42802-5)
Supplement: Supplementary file 1 — Supplementary Information [file 41467_2023_42802_MOESM1_ESM.pdf]

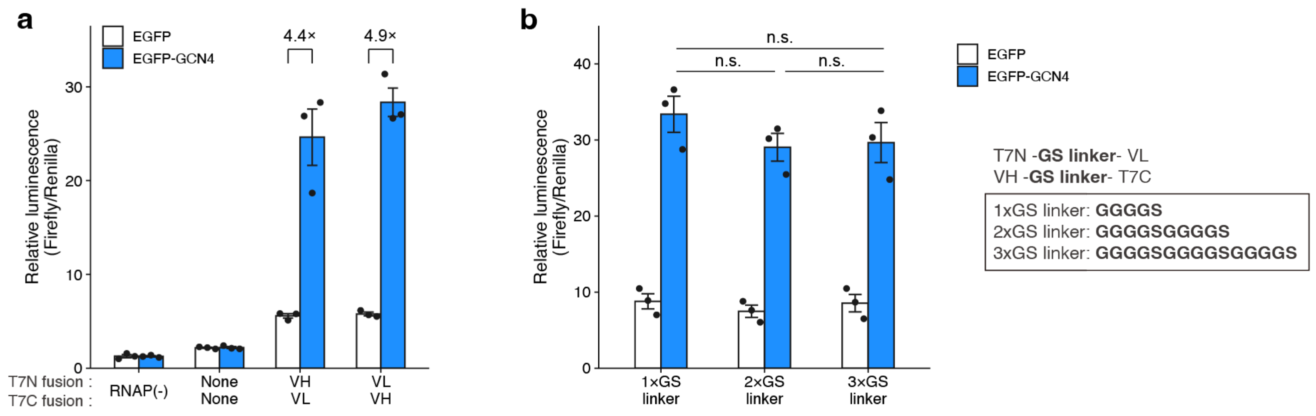

Supplementary Figure 1: Optimization of fusion pattern and linker length of TdRNAP.

a, Transcriptional response of the GCN4-dRNAP in 293FT cells. The fusion orientations of VH and VL domains to each half of the split T7 RNAP are shown below the graph. 50 of the induction plasmids were used. Each variable domain was fused with a 3xGS linker. RNAP(-), transfection with empty plasmid instead of split T7 RNAP fragments (T7N and T7C) **b**, Transcriptional response of the GCN4-dRNAP in 293FT cells. The linker length between variable domains and each half of the split T7 RNAP were varied as indicated in the figure. Values represent mean ± s.d. of n = 3 biological replicates (**a** and **b**) biological replicates. Statistical analysis by one-way ANOVA with Bonferroni correction (**b**), n.s., not significant (P > 0.05). Each P value is listed in Supplementary Table 5. Source data are provided as a Source Data file.

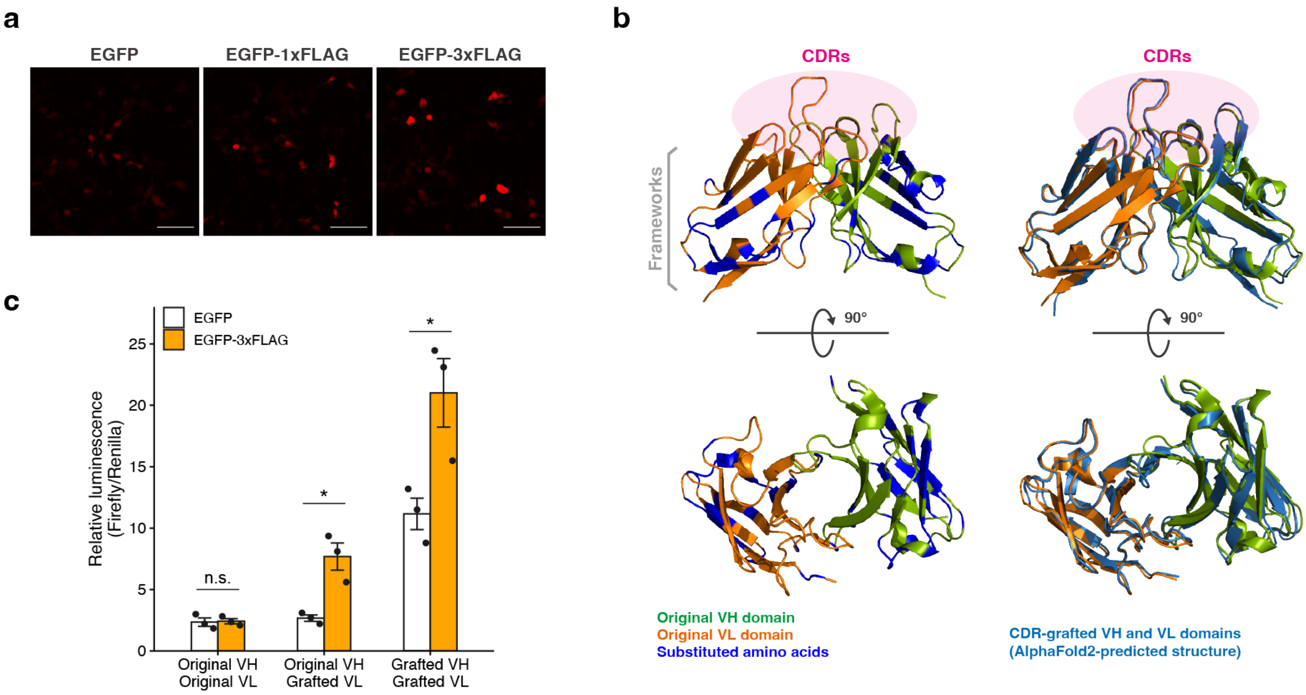

Supplementary Figure 2: Evaluation of FLAG-dRNAP with original and CDR-grafted variable domains.

a, Fluorescence images of 293FT cells transfected with FLAG-dRNAP consisting of original VH and VL domains of the anti-FLAG antibody. The transfected cells were induced with EGFP, EGFP-1xFLAG, or EGFP-3xFLAG. Scale bar, 100 μm. **b**, Structure of original and CDR-grafted variable domains of the anti-FLAG antibody. Original VH (green) and VL domains (orange) (PDB ID: 7BG1), substituted amino acids by CDR-grafting (blue), CDR-grafted VH and VL domains (skyblue) (AlphaFold2-predicted structure). **c**, Optimization of frameworks of VH and VL domains by CDR-grafting. CDRs for VH and VL domains of the anti-FLAG antibody were grafted to the trastuzumab frameworks (grafted VH and VL). Values represent mean ± s.e. of n = 3 biological replicates. Statistical analysis by unpaired two-tailed t-test, *$P < 0.05$, n.s., not significant ($P > 0.05$). Each P value is listed in Supplementary Table 5. Source data are provided as a Source Data file.

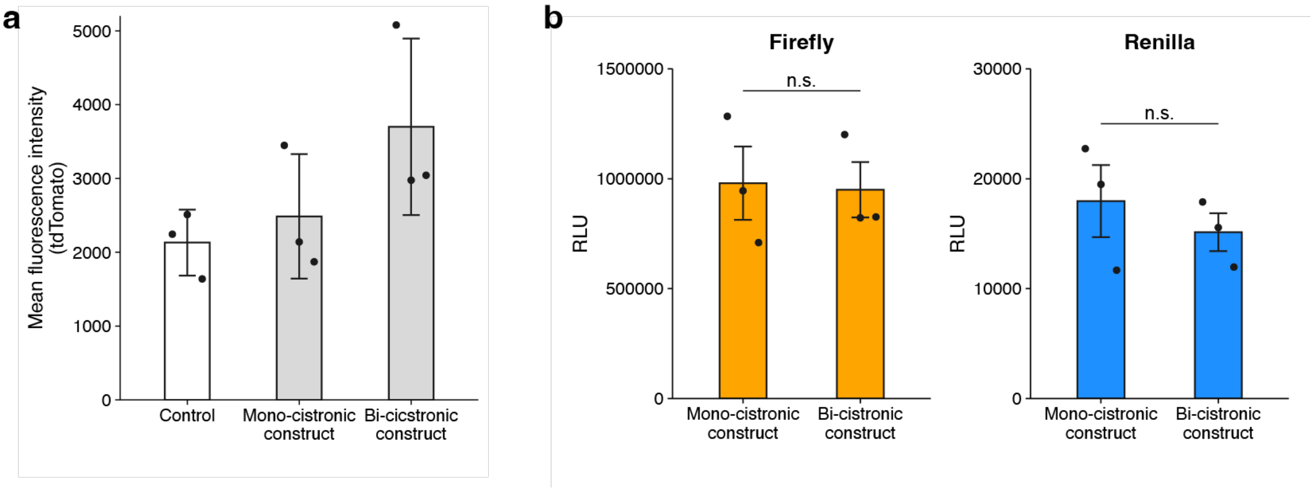

Supplementary Figure 3: Analysis of HCV IRES RNA-dependent transcriptional activation of HCV IRES RNA-dRNAP.

a, tdTomato mean fluorescence intensity measured by flow cytometry. The transfected 293FT cells were induced with empty plasmid (control), mono-cistronic constructs, or bi-cistronic construct. **b**, Comparison of luciferase expression levels from the mono-cistronic and bi-cistronic constructs. Values represent mean ± s.d. of n = 3 biological replicates (**a**) and mean ± s.e. of n = 3 biological replicates (**b**). Statistical analysis by unpaired two-tailed t-test (**b**), n.s., not significant ($P > 0.05$). Each P value is listed in Supplementary Table 5. Source data are provided as a Source Data file.

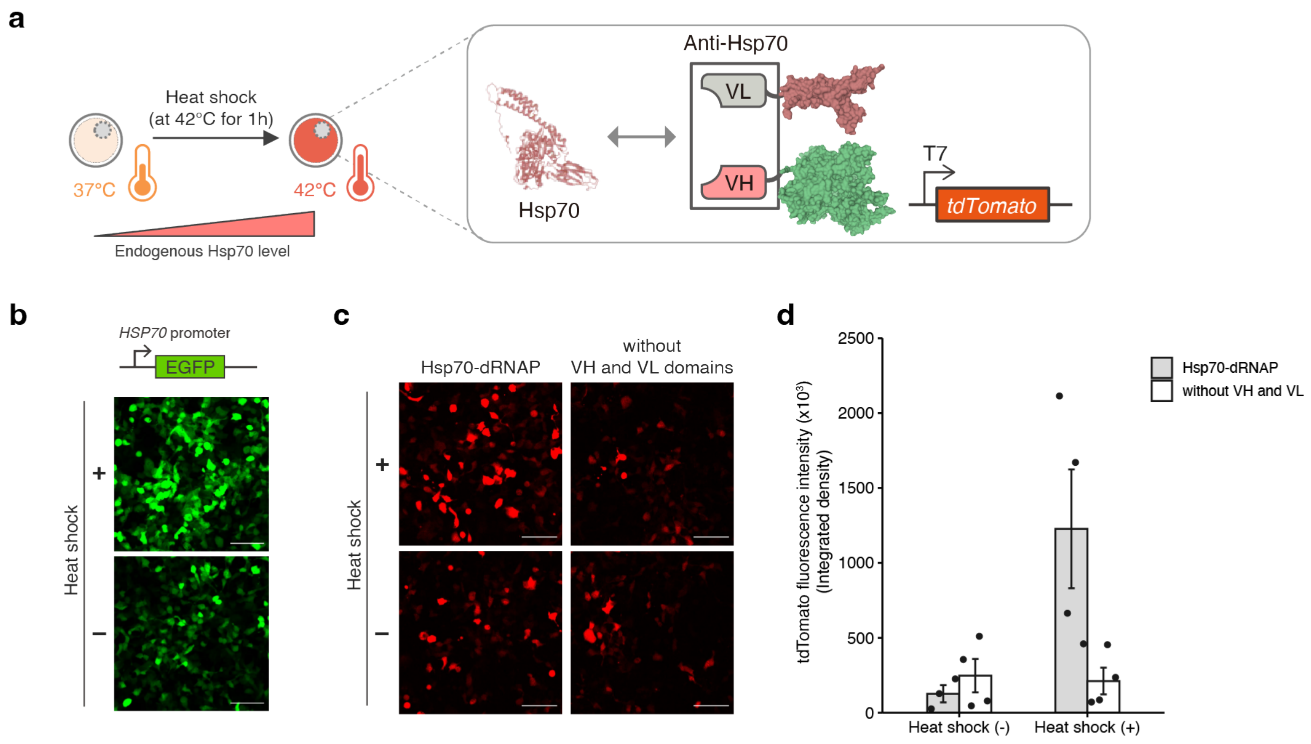

Supplementary Figure 4: Detection of endogenous Hsp70 using an Hsp70-dRNAP.

a, Design and application of Hsp70-dRNAP to monitor the changes in the endogenous Hsp70 expression level. The transfected 293FT cells were stimulated at 42℃ for 1 hour to increase the endogenous Hsp70 expression level. **b**, Validation of *HSP70* promoter activation after the heat shock stimulation using a plasmid encoding EGFP under *HSP70* promoter. Scale bar, 100 μm. **c**, Fluorescence images of the stimulated and unstimulated 293FT cells transfected with Hsp70-dRNAP or split RNAP without VH and VL domains. Scale bar, 100 μm. **d**, Comparison of the tdTomato fluorescence intensity between the stimulated and unstimulated 293FT cells transfected with Hsp70-dRNAP or split RNAP without VH and VL domains. The fluorescence intensity is quantified from the integrated density of tdTomato fluorescence. The protein structures were drawn with PDB data (PDB ID: 1QLN) and an AlphaFold-predicted structure (Hsp70: AF-P0DMV8-F1) (**a**). Values represent mean ± s.e. of n = 4 biological replicates (**d**). Grubbs' test was used to detect and exclude outliers (**d**), (α = 0.05). Source data are provided as a Source Data file.

Supplementary Table 1

Plasmid used in this study.

Plasmid name	Description	Figure
Split RNAP plasmids		
pCMV-T7N (d5-19)	N-terminal split T7 RNAP (d5-19)	Fig. S1a, S4c, S4d
pCMV-T7C	C-terminal split T7 RNAP	Fig. S1a, S4c, S4d
Target-dependent RNAP plasmids		
pCMV-T7N-VL (αGCN4)	N-terminal split T7 RNAP (d5-19) -GS linker- VL domain (Anti-GCN4 antibody)	Fig. 1c, 1d, 1e, 2e, 4b, 4d, 5c, 5d, Fig. S1a, S1b
pCMV-VH-T7C (αGCN4)	VH domain -GS linker- C-terminal split T7 RNAP (Anti-GCN4 antibody)	Fig. 1c, 1d, 2e, 4b, 5c, 5d, Fig. S1a, S1b
pCMV-VH-CGG RNAPc (αGCN4)	VH domain -GS linker- C-terminal split CGG RNAP (Anti-GCN4 antibody)	Fig. 4d
pCMV-T7N-VH (αGCN4)	N-terminal split T7 RNAP (d5-19) -GS linker- VH domain (Anti-GCN4 antibody)	Fig. S1a
pCMV-VL-T7C (αGCN4)	VL domain -GS linker- C-terminal split T7 RNAP (Anti-GCN4 antibody)	Fig. S1a
pCMV-VH-T7C (αGCN4, WT)	VH domain (WT) -GS linker- C-terminal split T7 RNAP (Anti-GCN4 antibody, omega-grafted framework)	Fig. 1e
pCMV-VH-T7C (αGCN4, GLW)	VH variant (GLW) -GS linker- C-terminal split T7 RNAP (Anti-GCN4 antibody, omega-grafted framework)	Fig. 1e
pCMV-VH-T7C (αGCN4, ALF)	VH variant (ALF) -GS linker- C-terminal split T7 RNAP (Anti-GCN4 antibody, omega-grafted framework)	Fig. 1e
pCMV-VH-T7C (αGCN4, GFA)	VH variant (GFA) -GS linker- C-terminal split T7 RNAP (Anti-GCN4 antibody, omega-grafted framework)	Fig. 1e
pCMV-T7N-VL (αFLAG, original)	N-terminal split T7 RNAP (d5-19) -GS linker- VL domain (Anti-FLAG antibody, original framework)	Fig. 2b, Fig. S2a, S2b
pCMV-VH-T7C (αFLAG, original)	VH domain -GS linker- C-terminal split T7 RNAP (Anti-FLAG antibody, original framework)	Fig. 2b, Fig. S2a, S2b
pCMV-T7N-VL (αFLAG, grafted)	N-terminal split T7 RNAP (d5-19) -GS linker- VL domain (Anti-FLAG antibody, trastuzumab framework)	Fig. 2b, 2c, 2e
pCMV-VH-T7C (αFLAG, grafted)	VH domain -GS linker- C-terminal split T7 RNAP (Anti-FLAG antibody, trastuzumab framework)	Fig. 2b, 2c, 2e
pCMV-T7N-VL (αEGFP)	N-terminal split T7 RNAP (d5-19) -GS linker- VL domain (Anti-EGFP antibody)	Fig. 2d, 2e, 4d
pCMV-VH-T7C (αEGFP)	VH domain -GS linker- C-terminal split T7 RNAP (Anti-EGFP antibody)	Fig. 2d, 2e, 4d
pCMV-T7N-VL (αHCV IRES)	N-terminal split T7 RNAP (d5-19) -GS linker- VL domain (Anti-HCV IRES RNA antibody)	Fig. 3b, 3c, Fig. S3a, S3b
pCMV-VH-T7C (αHCV IRES)	VH domain -GS linker- C-terminal split T7 RNAP (Anti-HCV IRES RNA antibody)	Fig. 3b, 3c, Fig. S3a, S3b
pCMV-T7N-VL (αFluorescein)	N-terminal split T7 RNAP (d5-19) -GS linker- VL domain (Anti-Fluorescein antibody)	Fig. 3d, 3e
pCMV-VH-T7C (αFluorescein)	VH domain -GS linker- C-terminal split T7 RNAP (Anti-Fluorescein antibody)	Fig. 3d, 3e
pCMV-T7N-VL (αHsp70)	N-terminal split T7 RNAP (d5-19) -GS linker- VL domain (Anti-Hsp70 antibody)	Fig. S4c, S4d
pCMV-VH-T7C (αHsp70)	VH domain -GS linker- C-terminal split T7 RNAP (Anti-Hsp70 antibody)	Fig. S4c, S4d
Induction plasmids		
pCMV-EGFP	EGFP	Fig. 1c, 1d, 1e, 2b, 2c, 2d, 2e, 4b, 4d, Fig. S1a, S1b, S2a
pCMV-EGFP-GCN4	EGFP -GS linker- GCN4 peptide	Fig. 1c, 1d, 1e, 2e, 4b, 4d, Fig. S1a, S1b
pCMV-EGFP-1xFLAG	EGFP -GS linker- 1xFLAG peptide	Fig. 2b, 2c, 2e, Fig. S2a
pCMV-EGFP-3xFLAG	EGFP -GS linker- 3xFLAG peptide	Fig. 2b, 2c, 2e, Fig. S2a

pCMV-Azami-Green	Azami-Green	Fig. 2d, 2e, 4d
pCMV-iRFP670	iRFP670	Fig. 4d
pCMV-iRFP670-GCN4	iRFP670 -GS linker- GCN4 peptide	Fig. 4d
pFR_HCV_xb	Bi-cistronic construct encoding luciferase (Firefly -HCV IRES- *Renilla*) under HSV TK promoter (Addgene_#11510)	Fig. 3b, 3c, Fig. S3a, S3b
pFluc	Mono-cistronic construct encoding firefly luciferase under HSV TK promoter	Fig. 3b, 3c, Fig. S3a, S3b
pRluc	Mono-cistronic construct encoding *Renilla* luciferase under HSV TK promoter	Fig. 3b, 3c, Fig. S3a, S3b
Reporter plasmids		
pT7-IRES2-iRFP670	EMCV IRES-driven iRFP670 under T7 promoter	Fig. 1c
pT7-IRES2-tdTomato	EMCV IRES-driven tdTomato under T7 promoter	Fig. 3b, 3c, 3d, Fig. S2a, S3a, S4c, S4d
pT7-IRES2-Fluc-pTK-Rluc	Construct encoding EMCV IRES-driven firefly luciferase under T7 promoter and *Renilla* luciferase under HSV TK promoter	Fig. 1d, 1e, 2b, 2c, 2d, 2e, 3e
pDual-IRES2-Fluc-pTK-Rluc	Construct encoding EMCV IRES-driven firefly luciferase under both CGG promoter and T7 promoter (CGG pro. - T7 pro. -EMCV IRES- firefly luciferase) and *Renilla* luciferase under HSV TK promoter	Fig. 4b
pT7-IRES2-CGG RNAP	EMCV IRES-driven CGG RNAP under T7 promoter	Fig. 4b
pT7-IRES2-Fluc	EMCV IRES-driven firefly luciferase under T7 promoter	Fig. 4d
pCGG-IRES2-Rluc	EMCV IRES-driven *Renilla* luciferase under CGG promoter	Fig. 4d
pT7-gRNA_EGFP	EGFP-targeting gRNA under T7 promoter	Fig. 5c, 5d
Plasmids used for cell line establishment and genome editing		
AAV-CMV-EGFP	EGFP gene under CMV promoter is flanked by homology arms targeting human *AAVS1* locus. (A splicing acceptor-T2A-PuroR cassette is located downstream of the left homology arm.)	Fig. 5b, 5c, 5d, 5e
AAV-CMV-EGFP-GCN4	EGFP-GCN4 gene under CMV promoter is flanked by homology arms targeting human *AAVS1* locus. (A splicing acceptor-T2A-PuroR cassette is located downstream of the left homology arm.)	Fig. 5b, 5c, 5d, 5e
hCas9	SpCas9 under CMV promoter (Addgene_##41815)	Fig. 5b, 5c, 5d, 5e
gRNA_AAVS1-T2	Human *AAVS1* locus-targeting gRNA under human U6 promoter (Addgene_#41818)	Fig. 5b
pU6-gRNA_EGFP	EGFP-targeting gRNA under human U6 promoter	Fig. 5e
Other plasmids		
pHSP70-EGFP	EGFP under human *HSP70* promoter	Fig. S4b
pUC19	Empty vector	Fig. 1d, 2c, 2d, 4b, 5c, 5d, 5e, Fig. S3a
pcDNA3.1	Empty vector	Fig. 1e, Fig. S1a

Supplementary Table 2

Amino acid sequences of individual proteins and peptides, used in the study.

T7N (d5-19) (N-terminal T7 RNAP fragment)
MNTINIAKNDFSDIELAAIPLNTLADHYGERSARGQLALEHESYEMGEARFRKMFECQLKAGKVADNAAAKPLITTLLPK<br>MIARINDWFEEVKAKRGRRPTAFKFLKEIKPEAVAYITIKTSLACLTSADNTTVQAVASAIGRTIEDEARFGRIRDLEAK<br>HFKKNVEEQLNKRVGHVYK
T7C (C-terminal T7 RNAP fragment)
KAFMQVVEADMLSKGLLGGEAWSSWHKEDSIHVGVRCIEMLIESTGMVSLHRQNAGVVGQDSETIELAPEYAEAIATRAG<br>ALAGISPMFQPCVVPPKPWTGITGGGYWANGRRPLALVRTHSKKALMRYEDVYMPEVYKAINIAQNTAWKINKKVLAVAN<br>VITKWKHCPVEDIPAIEREELPMKPEDIDMNPEALTAWKRAAAAVYRKDKARKSRRISLEFMLEQANKFANHKAIWFPYN<br>MDWRGRVYAVSMFNPQGNDMTKGLLTLAKGKPIGKEGYYWLKIHGANCAGVDKVPFPERIKFIEENHENIMACAKSPLEN<br>TWWAEQDSPFCFLAFCFEYAGVQHHGLSYNCSLPLAFDGSCSGIQHFSAMLRDEVGGRAVNLLPSETVQDIYGIVAKKVN<br>EILQADAINGTDNEVVTVTDENTGEISEKVKLGTKALAGQWLAYGVTRSVTKRSVMTLAYGSKEFGFRQQVLEDTIQPAI<br>DSGKGLMFTQPNQAAGYMAKLIWESVSVTVVAAVEAMNWLKSAAKLLAAEVKDKKTGEILRKRCAVHWVTPDGFPVWQEY<br>KKPIQTRLNLMFLGQFRLQPTINTNKDSEIDAHKQESGIAPNFVHSQDGSHLRKTVVWAHEKYGIESFALIHDSFGTIPA<br>DAANLFKAVRETMVDTYESCDVLADFYDQFADQLHESQLDKMPALPAKGNLNLRDILESDFAFA
CGG RNAPc (C-terminal CGG RNAP fragment)
KAFMQVVEADMLSKGLLGGEAWSSWHKEDSIHVGVRCIEMLIESTGMVSLHRQNAGVVGQDSETIELAPEYAEAIATRAG<br>ALAGISPMFQPCVVPPKPWTGITGGGYWANGRRPLALVRTHSKKALMRYEDVYMPEVYKAINIAQNTAWKINKKVLAVAN<br>VITKWKHCPVEDIPAIEREELPMKPEDIDMNPEALTAWKRAAAAVYRKDKARKSRRISLEFMLEQANKFANHKAIWFPYN<br>MDWRGRVYAVSMFNPQGNDMTKGLLTLAKGKPIGKEGYYWLKIHGANCAGVDKVPFPERIKFIEENHENIMACAKSPLEN<br>TWWAEQDSPFCFLAFCFEYAGVQHHGLSYNCSLPLAFDGSCSGIQHFSAMLRDEVGGRAVNLLPSETVQDIYGIVAKKVN<br>EILQADAINGTDNEVVTVTDENTGEISEKVKLGTKALAGQWLAYGVTRSVTKRSVMTLAYGSKEFGFRQQVLEDTIQPAI<br>DSGKGLMFTQPNQAAGYMAKLIWESVSVTVVAAVEAMNWLKSAAKLLAAEVKDKKTGEILRKRCAVHWVTPDGFPVWQEY<br>KKPIKTRVHIMFLGQFEMQPTINTNKDSEIDARKQVSGIAPNFVHSQDGSHLRKTVVWAHEKYGIESFALIHDSFGTIPA<br>DAANLFKAVRETMVDTYESCDVLADFYDQFADQLHESQLDKMPALPAKGNLNLRDILESDFAFA
CGG RNAP
MNTINIAKNDFSDIELAAIPFNTLADHYGERLAREQLALEHESYEMGEARFRKMFERQLKAGEVADNAAAKPLITTLLPK<br>MIARINDWFEEVKAKRGKRPTAFQFLQEIKPEAVAYITIKTTLACLTSADNTTVQAVASAIGRAIEDEARFGRIRDLEAK<br>HFKKNVEEQLNKRVGHVYKKAFMQVVEADMLSKGLLGGEAWSSWHKEDSIHVGVRCIEMLIESTGMVSLHRQNAGVVGQD<br>SETIELAPEYAEAIATRAGALAGISPMFQPCVVPPKPWTGITGGGYWANGRRPLALVRTHSKKALMRYEDVYMPEVYKAI<br>NIAQNTAWKINKKVLAVANVITKWKHCPVEDIPAIEREELPMKPEDIDMNPEALTAWKRAAAAVYRKDKARKSRRISLEF<br>MLEQANKFANHKAIWFPYNMDWRGRVYAVSMFNPQGNDMTKGLLTLAKGKPIGKEGYYWLKIHGANCAGVDKVPFPERIK<br>FIEENHENIMACAKSPLENTWWAEQDSPFCFLAFCFEYAGVQHHGLSYNCSLPLAFDGSCSGIQHFSAMLRDEVGGRAVN<br>LLPSETVQDIYGIVAKKVNEILQADAINGTDNEVVTVTDENTGEISEKVKLGTKALAGQWLAYGVTRSVTKRSVMTLAYG<br>SKEFGFRQQVLEDTIQPAIDSGKGLMFTQPNQAAGYMAKLIWESVSVTVVAAVEAMNWLKSAAKLLAAEVKDKKTGEILR<br>KRCAVHWVTPDGFPVWQEYKKPIKTRVHIMFLGQFEMQPTINTNKDSEIDARKQVSGIAPNFVHSQDGSHLRKTVVWAHE<br>KYGIESFALIHDSFGTIPADAANLFKAVRETMVDTYESCDVLADFYDQFADQLHESQLDKMPALPAKGNLNLRDILESDF<br>AFA
VL domain (Anti-GCN4 antibody)
GPDIVMTQSPSSLSASVGDRVTITCRSSTGAVTTSNYASWVQEKPGKLFKGLIGGTNNRAPGVPSRFSGSLIGDKATLTI<br>SSLQPEDFATYFCALWYSNHWVFGQGTKVELKR
VH domain (Anti-GCN4 antibody)
EVKLLESGGGLVQPGGSLKLSCAVSGFSLTDYGVNWVRQAPGRGLEWIGVIWGDGITDYNSALKDRFIISKDNGKNTVYL<br>QMSKVRSDDTALYYCVTGLFDYWGQGTLVTVSS
VH domain (WT), (Anti-GCN4 antibody, omega-grafted framework)
EVKLLESGGGLVQPGGSLKLSCAVSGFSLTDYGVNWVRQAPGRGLEWIGVIWGDGITDYNSALKDRFIISKDDCENTVYL<br>QMSKVRSDDTALYYCVTGLFDYWGQGTLVTVSS
VH domain (GLW), (Anti-GCN4 antibody, omega-grafted framework)
EVKLLESGGGLVQPGGSLKLSCAVSGFSLTDYGVNWVRQAPGRGLEWIGVIWGDGITDYNSALKDRFIISKDDCENTVYL<br>QMSKVRSDDTALYYCVTGLWDYWGQGTLVTVSS
VH domain (ALF), (Anti-GCN4 antibody, omega-grafted framework)
EVKLLESGGGLVQPGGSLKLSCAVSGFSLTDYGVNWVRQAPGRGLEWIGVIWGDGITDYNSALKDRFIISKDDCENTVYL<br>QMSKVRSDDTALYYCVTALFDYWGQGTLVTVSS
VH domain (GFA), (Anti-GCN4 antibody, omega-grafted framework)
EVKLLESGGGLVQPGGSLKLSCAVSGFSLTDYGVNWVRQAPGRGLEWIGVIWGDGITDYNSALKDRFIISKDDCENTVYL<br>QMSKVRSDDTALYYCVTGFADYWGQGTLVTVSS
VL domain (Anti-FLAG antibody, original framework)
DVLMTQIPLSLPVSLGDQASISCRSSQSIVHRNGNTYLEWYLLKPGQSPKLLIYKVSNRFSGVPDRFSGSGSGTDFTLKI<br>SRVEAEDLGVYYCFQGSHVPYTFGGGTKLEIKR
VL domain (Anti-FLAG antibody, trastuzumab framework)

DIQMTQSPSSLSASVGDRVTITCRSSQSIVHRNGNTYLEWYQQKPGKAPKLLIYKVSNRFSGVPSRFSGSGSGTDFTLTISSLQPEDFATYYCFQGSHVPYTFGQGTKVEIKR
VH domain (Anti-FLAG antibody, original framework)
QVQLQQSAAELARPGASVKMSCKASGYSFTTYTIHWVKQRPGQGLEWIGYINPSSGYAAYNQNFKDETTLTADPSSSTAYMELNSLTSEDSAVYYCAREKFYGYDYWGQGATLTVSS
VH domain (Anti-FLAG antibody, trastuzumab framework)
EVQLLESGGGLVQPGGSLRLSCAASGYSFTTYTIHWVRQAPGKGLEWIGYINPSSGYAAYADSVKGRFTISRDNSKNTLYLQMNSLRAEDTAVYYCAREKFYGYDYWGQGTLVTVSS
VL domain (Anti-EGFP antibody)
STDIQMTQSPSSLSASVGDRVTITCRASQSISSYLNWYQQKPGKAPKLLIYYASYLQSGVPSRFSGSGSGTDFTLTISSLQPEDFATYYCQQTAAGPSTFGQGTKVEIKR
VH domain (Anti-EGFP antibody)
EVQLLESGGGLVQPGGSLRLSCAASGFTFSSYAMSWVRQAPGKGLEWVSYISGTGNTTAYADSVKGRFTISRDNSKNTLYLQMNSLRAEDTAVYYCAKDTNYFDYWGQGTLVTVSS
VL domain (Anti-HCV IRES RNA antibody)
DIQMTQSPSSLSASVGDRVTITCRASQSVSSAVAWYQQKPGKAPKLLIYSASSLYSGVPSRFSGSGSGTDFTLTISSLQPEDFATYYCQQSSYYPSTFGQGTKVEIKR
VH domain (Anti-HCV IRES RNA antibody)
EVQLLESGGGLVQPGGSLRLSCAASGFYISSYSIHWVRQAPGKGLEWVASIYPSYGYTSYADSVKGRFTISADNSKNTLYLQMNSLRAEDTAVYYCARRYRSYYSRYGFDYWGQGTLVTVSS
VL domain (Anti-Fluorescein antibody)
DIQMTQSPSSLSASVGDRVTITCRSSQSLVHSNGNTYLRWYQQKPGKAPKLLIYKVSNRVSGVPSRFSGSGSGTDFTLTISSLQPEDFATYYCSQSTHVPWTFGQGTKVEIKR
VH domain (Anti-Fluorescein antibody)
EVQLLESGGGLVQPGGSLRLSCAASGFTFGHYWMNWVRQAPGKGLEWVAQFRNKPYNYETYYADSVKGRFTISRDNSKNTLYLQMNSLRAEDTAVYYCTGASYGMEYWGQGTLVTVSS
VL domain (Anti-Hsp70 antibody)
DIQMTQSPSSLSASVGDRVTITCRSSTGAVTTSNYANWYQQKPGKAPKLLIGGTNNRAPGVPSRFSGSGSGTDFTLTISSLQPEDFATYYCALWYSNHLVFGQGTKVEIKR
VH domain (Anti-Hsp70 antibody)
EVQLLESGGGLVQPGGSLRLSCAASGFSLSRNSVHWVRQAPGKGLEWLGMIWGGGSTDYADSVKGRFTISRDNSKNTLYLQMNSLRAEDTAVYYCARNGGYDVFDYWGQGTLVTVSS
EGFP
MVSKGEELFTGVVPILVELDGDVNGHKFSVSGEGEGDATYGKLTLKFICTTGKLPVPWPTLVTTLTYGVQCFSRYPDHMKQHDFFKSAMPEGYVQERTIFFKDDGNYKTRAEVKFEGDTLVNRIELKGIDFKEDGNILGHKLEYNYNSHNVYIMADKQKNGIKVNFKIRHNIEDGSVQLADHYQQNTPIGDGPVLLPDNHYLSTQSALSKDPNEKRDHMVLLEFVTAAGITLGMDELYK
Azami-Green
MGSVSVIKPEMKIKLCMRGTVNGHNFVIEGEGKGNPYEGTQILDLNVTEGAPLPFAYDILTTVFQYGNRAFTKYPADIQDYFKQTFPEGYHWERSMTYEDQGICTATSNISMRGDCFFYDIRFDGTNFPPNGPVMQKKTLKWEPSTEKMYVEDGVLKGDVNMRLLLEGGGHYRCDFKTTYKAKKEVRLPDAHKIDHRIEILKHDKDYNKVKLYENAVARYSMLPSQAK
iRFP670
MARKVDLTSCDREPIHIPGSIQPCGCLLACDAQAVRITRITENAGAFFGRETPRVGELLADYFGETEAHALRNALAQSSDPKRPALIFGWRDGLTGRTFDISLHRHDGTSIIEFEPAAAEQADNPLRLTRQIIARTKELKSLEEMAARVPRYLQAMLGYHRVMLYRFADDGSGMVIGEAKRSDLESFLGQHFPASLVPQQARLLYLKNAIRVVSDSRGISSRIVPEHDASGAALDLSFAHLRSISPCHLEFLRNMGVSASMSLSIIIDGTLWGLIICHHYEPRAVPMAQRVAAEMFADFLSLHFTAAHHQ
GCN4 peptide
EELLSKNYHLENEVARLKK
1xFLAG peptide
DYKDDDDK
3xFLAG peptide
DYKDHDGDYKDHDIDYKDDDDK
Firefly luciferase
MEDAKNIKKGPAPFYPLEDGTAGEQLHKAMKRYALVPGTIAFTDAHIEVDITYAEYFEMSVRLAEAMKRYGLNTNHRIVVCSENSLQFFMPVLGALFIGVAVAPANDIYNERELLNSMGISQPTVVFVSKKGLQKILNVQKKLPIIQKIIIMDSKTDYQGFQSMYTFVTSHLPPGFNEYDFVPESFDRDKTIALIMNSSGSTGLPKGVALPHRTACVRFSHARDPIFGNQIIPDTAILSVVPFHHGFGMFTTLGYLICGFRVVLMYRFEEELFLRSLQDYKIQSALLVPTLFSFFAKSTLIDKYDLSNLHEIASGGAPLSKEVGEAVAKRFHLPGIRQGYGLTETTSAILITPEGDDKPGAVGKVVPFFEAKVVDLDTGKTLGVNQRGELCVRGPMIMSGYVNNPEATNALIDKDGWLHSGDIAYWDEDEHFFIVDRLKSLIKYKGYQVAPAELESILLQHPNIFDAGVAGLPDDDAGELPAAVVVLEHGKTMTEKEIVDYVASQVTTAKKLRGGVVFVDEVPKGLTGKLDARKIREILIKAKKGGKIAV
Renilla luciferase
MTSKVYDPEQRKRMITGPQWWARCKQMNVLDSFINYYDSEKHAENAVIFLHGNAASSYLWRHVVPHIEPVARCIIPDLIG

MGKSGKSGNGSYRLLDHYKYLTAWFELLNLPKKIIFVGHDWGACLAFHYSYEHQDKIKAIVHAESVVDVIESWDEWPDIE
EDIALIKSEEGEKMVLENNFFVETMLPSKIMRKLEPEEFAAYLEPFKEKGEVRRPTLSWPREIPLVKGGKPDVVQIVRNY
NAYLRASDDLPKMFIESDPGFFSNAIVEGAKKFPNTEFVKVKGLHFSQEDAPDEMGKYIKSFVERVLKNEQ

tdTomato

MVSKGEEVIKEFMRFKVRMEGSMNGHEFEIEGEGEGRPYEGTQTAKLKVTKGGPLPFAWDILSPQFMYGSKAYVKHPADI
PDYKKLSFPEGFKWERVMNFEDGGLVTVTQDSSLQDGTLIYKVKMRGTNFPPDGPVMQKKTMGWEASTERLYPRDGVLKG
EIHQALKLKDGGHYLVEFKTIYMAKKPVQLPGYYYVDTKLDITSHNEDYTIVEQYERSEGRHHLFLGHGTGSTGSGSSGT
ASSEDNNMAVIKEFMRFKVRMEGSMNGHEFEIEGEGEGRPYEGTQTAKLKVTKGGPLPFAWDILSPQFMYGSKAYVKHPA
DIPDYKKLSFPEGFKWERVMNFEDGGLVTVTQDSSLQDGTLIYKVKMRGTNFPPDGPVMQKKTMGWEASTERLYPRDGVL
KGEIHQALKLKDGGHYLVEFKTIYMAKKPVQLPGYYYVDTKLDITSHNEDYTIVEQYERSEGRHHLFLYGMDELYK

SpCas9

MDKKYSIGLDIGTNSVGWAVITDEYKVPSKKFKVLGNTDRHSIKKNLIGALLFDSGETAEATRLKRTARRRYTRRKNRIC
YLQEIFSNEMAKVDDSFFHRLEESFLVEEDKKHERHPIFGNIVDEVAYHEKYPTIYHLRKKLVDSTDKADLRLIYLALAH
MIKFRGHFLIEGDLNPDNSDVDKLFIQLVQTYNQLFEENPINASGVDAKAILSARLSKSRRLENLIAQLPGEKKNGLFGN
LIALSLGLTPNFKSNFDLAEDAKLQLSKDTYDDDLDNLLAQIGDQYADLFLAAKNLSDAILLSDILRVNTEITKAPLSAS
MIKRYDEHHQDLTLLKALVRQQLPEKYKEIFFDQSKNGYAGYIDGGASQEEFYKFIKPILEKMDGTEELLVKLNREDLLR
KQRTFDNGSIPHQIHLGELHAILRRQEDFYPFLKDNREKIEKILTFRIPYYVGPLARGNSRFAWMTRKSEETITPWNFEE
VVDKGASAQSFIERMTNFDKNLPNEKVLPKHSLLYEYFTVYNELTKVKYVTEGMRKPAFLSGEQKKAIVDLLFKTNRKVT
VKQLKEDYFKKIECFDSVEISGVEDRFNASLGTYHDLLKIIKDKDFLDNEENEDILEDIVLTLTLFEDREMIEERLKTYA
HLFDDKVMKQLKRRRYTGWGRLSRKLINGIRDKQSGKTILDFLKSDGFANRNFMQLIHDDSLTFKEDIQKAQVSGQGDSL
HEHIANLAGSPAIKKGILQTVKVVDELVKVMGRHKPENIVIEMARENQTTQKGQKNSRERMKRIEEGIKELGSQILKEHP
VENTQLQNEKLYLYYLQNGRDMYVDQELDINRLSDYDVDHIVPQSFLKDDSIDNKVLTRSDKNRGKSDNVPSEEVVKKMK
NYWRQLLNAKLITQRKFDNLTKAERGGLSELDKAGFIKRQLVETRQITKHVAQILDSRMNTKYDENDKLIREVKVITLKS
KLVSDFRKDFQFYKVREINNYHHAHDAYLNAVVGTALIKKYPKLESEFVYGDYKVYDVRKMIAKSEQEIGKATAKYFFYS
NIMNFFKTEITLANGEIRKRPLIETNGETGEIVWDKGRDFATVRKVLSMPQVNIVKKTEVQTGGFSKESILPKRNSDKLI
ARKKDWDPKKYGGFDSPTVAYSVLVVAKVEKGKSKKLKSVKELLGITIMERSSFEKNPIDFLEAKGYKEVKKDLIIKLPK
YSLFELENGRKRMLASAGELQKGNELALPSKYVNFLYLASHYEKLKGSPEDNEQKQLFVEQHKHYLDEIIEQISEFSKRV
ILADANLDKVLSAYNKHRDKPIREQAENIIHLFTLTNLGAPAAFKYFDTTIDRKRYTSTKEVLDATLIHQSITGLYETRI
DLSQLGGD

Supplementary Table 3

Nucleotide sequences of key genes and regulatory elements, used in the study.

T7 promoter
TAATACGACTCACTATAGGG
CGG promoter
TAATACCGGTCACTATAGGG
HCV IRES
CTCCCCTGTGAGGAACTACTGTCTTCACGCAGAAAGCGTCTAGCCATGGCGTTAGTATGAGAGTCGTGCAGCCTCCAGGA<br>CCCCCCCTCCCGGGAGAGCCATAGTGGTCTGCGGAACCGGTGAGTACACCGGAATTGCCAGGACGACCGGGTCCTTTCTT<br>GGATCAACCCGCTCAATGCCTGGAGATTTGGGCGTGCCCCCGCAAGACTGCTAGCCGAGTAGTGTTGGGTCGCGAAAGGC<br>CTTGTGGTACTGCCTGATAGGGTGCTTGCGAGTGCCCCGGGAGGTCTCGTAGACCGTGCACCATGAGCACGAATCCTAAA<br>CCTCAAAGAAAAA
EGFP-targeting gRNA
GCACGGGCAGCTTGCCGGGTTTTAGAGCTAGAAATAGCAAGTTAAAATAAGGCTAGTCCGTTATCAACTTGAAAAAGTGG<br>CACCGAGTCGGTGCT
EGFP (gRNA target sequence is underlined)
ATGGTGAGCAAGGGCGAGGAGCTGTTCACCGGGGTGGTGCCCATCCTGGTCGAGCTGGACGGCGACGTAAACGGCCACAA<br>GTTCAGCGTGTCCGGCGAGGGCGAGGGCGATGCCACCTACGGCAAGCTGACCCTGAAGTTCATCTGCACCA<u>CCGGCAAGC</u><br><u>TGCCCGTGC</u>CCTGGCCCACCCTCGTGACCACCCTGACCTACGGCGTGCAGTGCTTCAGCCGCTACCCCGACCACATGAAG<br>CAGCACGACTTCTTCAAGTCCGCCATGCCCGAAGGCTACGTCCAGGAGCGCACCATCTTCTTCAAGGACGACGGCAACTA<br>CAAGACCCGCGCCGAGGTGAAGTTCGAGGGCGACACCCTGGTGAACCGCATCGAGCTGAAGGGCATCGACTTCAAGGAGG<br>ACGGCAACATCCTGGGGCACAAGCTGGAGTACAACTACAACAGCCACAACGTCTATATCATGGCCGACAAGCAGAAGAAC<br>GGCATCAAGGTGAACTTCAAGATCCGCCACAACATCGAGGACGGCAGCGTGCAGCTCGCCGACCACTACCAGCAGAACAC<br>CCCCATCGGCGACGGCCCCGTGCTGCTGCCCGACAACCACTACCTGAGCACCCAGTCCGCCCTGAGCAAAGACCCCAACG<br>AGAAGCGCGATCACATGGTCCTGCTGGAGTTCGTGACCGCCGCCGGGATCACTCTCGGCATGGACGAGCTGTACAAG
Human *HSP70* promoter
CCCTGTCCCCTCCAGTGAATCCCAGAAGACTCTGGAGAGTTCTGAGCAGGGGGCGGCACTCTGGCCTCTGATTGGTCCAA<br>GGAAGGCTGGGGGGCAGGACGGGAGGCGAAAACCCTGGAATATTCCCGACCTGGCAGCCTCATCGAGCTCGGTGATTGGC<br>TCAGAAGGGAAAAGGCGGGTCTCCGTGACGACTTATAAAAGCCCAGGGGCAAGCGGTCCGG

Supplementary Table 4

Plasmids mixtures for transfection, used in this study.

Figure	Plasmid	Amount (ng)	Total (ng)
Fig. 1c	pCMV-T7N-VL (αGCN4)<br>pCMV-VH-T7C (αGCN4)<br>pCMV-EGFP<br>pCMV-EGFP-GCN4<br>pT7-IRES2-iRFP670	15<br>35<br>0 or 50<br>0 or 50<br>50	150
Fig. 1d	pCMV-T7N-VL (αGCN4)<br>pCMV-VH-T7C (αGCN4)<br>pCMV-EGFP<br>pCMV-EGFP-GCN4<br>pT7-IRES2-Fluc-pTK-Rluc<br>pUC19	15<br>35<br>0, 10, 20, 40, 60, or 80<br>0, 10, 20, 40, 60, or 80<br>50<br>up to 180	180
Fig. 1e	pCMV-T7N-VL (αGCN4)<br>pCMV-VH-T7C (αGCN4), (WT, GLW, ALF, or GFA)<br>pCMV-EGFP<br>pCMV-EGFP-GCN4<br>pT7-IRES2-Fluc-pTK-Rluc	15<br>35<br>0 or 50<br>0 or 50<br>50	150
Fig. 2b	pCMV-T7N-VL (αFLAG), (original or grafted)<br>pCMV-VH-T7C (αFLAG), (original or grafted)<br>pCMV-EGFP<br>pCMV-EGFP-3xFLAG<br>pT7-IRES2-Fluc-pTK-Rluc	25<br>25<br>0 or 100<br>0 or 100<br>50	200
Fig. 2c	pCMV-T7N-VL (αFLAG), (original or grafted)<br>pCMV-VH-T7C (αFLAG), (original or grafted)<br>pCMV-EGFP<br>pCMV-EGFP-1xFLAG<br>pCMV-EGFP-3xFLAG<br>pT7-IRES2-Fluc-pTK-Rluc<br>pUC19	25<br>25<br>0, 50, 100, or 200<br>0, 50, 100, or 200<br>0, 50, 100, or 200<br>50<br>up to 300	300
Fig. 2d	pCMV-T7N-VL (αEGFP)<br>pCMV-VH-T7C (αEGFP)<br>pCMV-Azami-Green<br>pCMV-EGFP<br>pT7-IRES2-Fluc-pTK-Rluc<br>pUC19	25<br>25<br>0, 50, 100, or 200<br>0, 50, 100, or 200<br>50<br>up to 300	300
Fig. 2e (left)	pCMV-T7N-VL (αGCN4)<br>pCMV-VH-T7C (αGCN4)<br>pCMV-Azami-Green<br>pCMV-EGFP<br>pCMV-EGFP-GCN4<br>pCMV-EGFP-3xFLAG<br>pT7-IRES2-Fluc-pTK-Rluc	15<br>35<br>0 or 100<br>0 or 100<br>0 or 100<br>0 or 100<br>50	200
Fig. 2e (middle)	pCMV-T7N-VL (αFLAG, original)<br>pCMV-VH-T7C (αFLAG, original)<br>pCMV-Azami-Green<br>pCMV-EGFP<br>pCMV-EGFP-GCN4<br>pCMV-EGFP-3xFLAG<br>pT7-IRES2-Fluc-pTK-Rluc	25<br>25<br>0 or 100<br>0 or 100<br>0 or 100<br>0 or 100<br>50	200
Fig. 2e (right)	pCMV-T7N-VL (αEGFP)<br>pCMV-VH-T7C (αEGFP)<br>pCMV-Azami-Green<br>pCMV-EGFP<br>pCMV-EGFP-GCN4<br>pCMV-EGFP-3xFLAG<br>pT7-IRES2-Fluc-pTK-Rluc	25<br>25<br>0 or 100<br>0 or 100<br>0 or 100<br>0 or 100<br>50	200
Fig. 3b (left)<br>Fig. S3a<br>Fig. S3b	pCMV-T7N-VL (αHCV IRES)<br>pCMV-VH-T7C (αHCV IRES)<br>pFluc	25<br>25<br>150	300

	pRluc	50	
	pT7-IRES2-tdTomato	50	
Fig. 3b (right)	pCMV-T7N-VL (αHCV IRES)	25	300
Fig. S3a	pCMV-VH-T7C (αHCV IRES)	25	
Fig. S3b	pFR_HCV IRES_xb	200	
	pT7-IRES2-tdTomato	50	
Fig. 3d	pCMV-T7N-VL (αFluorescein)	25	100
	pCMV-VH-T7C (αFluorescein)	25	
	pT7-IRES2-tdTomato	50	
Fig. 3e	pCMV-T7N-VL (αFluorescein)	25	100
	pCMV-VH-T7C (αFluorescein)	25	
	pT7-IRES2-Fluc-pTK-Rluc	50	
Fig. 4b	pCMV-T7N-VL (αGCN4)	15	150
	pCMV-VH-T7C (αGCN4)	35	
	pCMV-EGFP	0 or 10	
	pCMV-EGFP-GCN4	0 or 10	
	pT7-IRES2-CGG RNAP	0-40	
	pDual-IRES2-Fluc-pTK-Rluc	50	
	pUC19	up to 150	
Fig. 4d	pCMV-T7N-VL (αGCN4)	12.5	400
	pCMV-VH-CGG RNAPc (αGCN4)	12.5	
	pCMV-T7N-VL (αEGFP)	50	
	pCMV-VH-T7C (αEGFP)	50	
	pCMV-Azami-Green	0 or 100	
	pCMV-EGFP	0 or 100	
	pCMV-iRFP670	0 or 25	
	pCMV-iRFP670-GCN4	0 or 25	
	pT7-IRES2-Fluc	50	
	pCGG-IRES2-Rluc	100	
Fig. 5b	AAV-CMV-EGFP	0 or 500	1500
	AAV-CMV-EGFP-GCN4	0 or 500	
	hCas9	500	
	gRNA_AAVS1-T2	500	
Fig. 5c (top)	pCMV-T7N-VL (αGCN4)	15	350
Fig. 5d	pCMV-VH-T7C (αGCN4)	35	
	hCas9	200	
	pT7-gRNA_EGFP	0 or 100	
	pUC19	0 or 100	
Fig. 5c (bottom)	pCMV-T7N-VL (αGCN4)	15	550
	pCMV-VH-T7C (αGCN4)	35	
	hCas9	400	
	pT7-gRNA_EGFP	0 or 100	
	pUC19	0 or 100	
Fig. 5e	pCMV-T7N-VL (αGCN4)	15	350
	pCMV-VH-T7C (αGCN4)	35	
	hCas9	200	
	pU6-gRNA_EGFP	0 or 100	
	pUC19	0 or 100	
Fig. 1e (RNAP-)	pcDNA3.1	50	150
Fig. S1a (RNAP-)	pCMV-EGFP	0 or 50	
	pCMV-EGFP-GCN4	0 or 50	
	pT7-IRES2-Fluc-pTK-Rluc	50	
Fig. S1a (None)	pCMV-T7N (d5-19)	15	150
	pCMV-T7C	35	
	pCMV-EGFP	0 or 50	
	pCMV-EGFP-GCN4	0 or 50	
	pT7-IRES2-Fluc-pTK-Rluc	50	
Fig. S1a (VH_VL)	pCMV-T7N-VH (αGCN4)	15	150
	pCMV-VL-T7C (αGCN4)	35	
	pCMV-EGFP	0 or 50	
	pCMV-EGFP-GCN4	0 or 50	
	pT7-IRES2-Fluc-pTK-Rluc	50	

Fig. S1a (VL_VH)	pCMV-T7N-VL (αGCN4)<br>pCMV-VH-T7C (αGCN4)<br>pCMV-EGFP<br>pCMV-EGFP-GCN4<br>pT7-IRES2-Fluc-pTK-Rluc	15<br>35<br>0 or 50<br>0 or 50<br>50	150
Fig. S1b	pCMV-T7N-VL (αGCN4) (1x, 2x, or 3x GS linker)<br>pCMV-VH-T7C (αGCN4) (1x, 2x, or 3x GS linker)<br>pCMV-EGFP<br>pCMV-EGFP-GCN4<br>pT7-IRES2-Fluc-pTK-Rluc	15<br>35<br>0 or 50<br>0 or 50<br>50	150
Fig. S2a	pCMV-T7N-VL (αFLAG), (original or grafted)<br>pCMV-VH-T7C (αFLAG), (original or grafted)<br>pCMV-EGFP<br>pCMV-EGFP-3xFLAG<br>pT7-IRES2-tdTomato	25<br>25<br>0 or 100<br>0 or 100<br>50	200
Fig. S3a (control)	pCMV-T7N-VL (αHCV IRES)<br>pCMV-VH-T7C (αHCV IRES)<br>pUC19<br>pT7-IRES2-tdTomato	25<br>25<br>200<br>50	300
Fig. S4b	pHSP70-EGFP	100	100
Fig. S4c<br>Fig. S4d	pCMV-T7N-VL (αHsp70)<br>pCMV-VH-T7C (αHsp70)<br>pCMV-T7N (d5-19)<br>pCMV-T7C<br>pT7-IRES2-tdTomato	0 or 100<br>0 or 100<br>0 or 100<br>0 or 100<br>50	250

Supplementary Table 5

Statistics and *P*-values.

Figure	Condition	Condition 1	Condition 2	Statistical analysis	p-value
Fig. 2b	Original VL domain	EGFP	EGFP-3xFLAG	unpaired two-tailed t-test	0.8539
	CDR-grafted VL domain	EGFP	EGFP-3xFLAG		0.01144
Fig. 2b	Anti-GCN4	Azami-Green	EGFP-GCN4	one-way ANOVA with Bonferroni correction	0.01
	Anti-GCN4	EGFP	EGFP-GCN4		0.005
	Anti-GCN4	EGFP-3xFLAG	EGFP-GCN4		0.005
	Anti-FLAG	Azami-Green	EGFP-3xFLAG		0.036
	Anti-FLAG	EGFP	EGFP-3xFLAG		0.036
	Anti-FLAG	EGFP-GCN4	EGFP-3xFLAG		0.04
	Anti-EGFP	Azami-Green	EGFP		0.03
	Anti-EGFP	EGFP-GCN4	EGFP		0.009
	Anti-EGFP	EGFP-3xFLAG	EGFP		0.007
Fig. 3c	-	Mono-cistronic	Bi-cistronic	unpaired two-tailed t-test	0.01394
Fig. 3e	Fluorescein treatment	0 μg/ml	50 μg/ml	one-way ANOVA with Dunnett's multiple comparison test	0.0843
	Fluorescein treatment	0 μg/ml	100 μg/ml		0.044
Fig. 5d	T7-gRNA(-)	EGFP cell lines	EGFP-GCN4 cell lines	unpaired two-tailed t-test	0.1131
	T7-gRNA(+)	EGFP cell lines	EGFP-GCN4 cell lines		0.001213
Fig. 5e	U6-gRNA(-)	EGFP cell lines	EGFP-GCN4 cell lines	unpaired two-tailed t-test	0.6405
	U6-gRNA(+)	EGFP cell lines	EGFP-GCN4 cell lines		0.6249
Fig. S1b	-	1xGS linker	2xGS-linker	one-way ANOVA with Bonferroni correction	0.672
	-	1xGS linker	3xGS linker		1
	-	2xGS linker	3xGS linker		1
Fig. S2c	Original VH + Original VL	EGFP	EGFP-3xFLAG	unpaired two-tailed t-test	0.8539
	Original VH + Grafted VL	EGFP	EGFP-3xFLAG		0.01144
	Grafted VH + Grafted VL	EGFP	EGFP-3xFLAG		0.03259
Fig. S3b	Firefly luciferase	Mono-cistronic	Bi-cistronic	unpaired two-tailed t-test	0.7219
	Renilla luciferase	Mono-cistronic	Bi-cistronic		0.4302
