# Supplementary material for: Target-dependent RNA polymerase as universal platform for gene expression control in response to intracellular molecules
Source: Nat Commun. 2023 Nov 17;14:7256. doi: 10.1038/s41467-023-42802-5 (PMC10656481; doi:10.1038/s41467-023-42802-5)
Supplement: Supplementary file 2 — Reporting Summary [file 41467_2023_42802_MOESM2_ESM.pdf]

nature portfolio

Corresponding author(s): Hirohide Saito

Last updated by author(s): Oct 20, 2023

Reporting Summary

Nature Portfolio wishes to improve the reproducibility of the work that we publish. This form provides structure for consistency and transparency in reporting. For further information on Nature Portfolio policies, see our Editorial Policies and the Editorial Policy Checklist.

Statistics

For all statistical analyses, confirm that the following items are present in the figure legend, table legend, main text, or Methods section.

n/a	Confirmed	
☐	☒	The exact sample size (*n*) for each experimental group/condition, given as a discrete number and unit of measurement
☐	☒	A statement on whether measurements were taken from distinct samples or whether the same sample was measured repeatedly
☐	☒	The statistical test(s) used AND whether they are one- or two-sided<br>*Only common tests should be described solely by name; describe more complex techniques in the Methods section.*
☒	☐	A description of all covariates tested
☐	☒	A description of any assumptions or corrections, such as tests of normality and adjustment for multiple comparisons
☐	☒	A full description of the statistical parameters including central tendency (e.g. means) or other basic estimates (e.g. regression coefficient) AND variation (e.g. standard deviation) or associated estimates of uncertainty (e.g. confidence intervals)
☐	☒	For null hypothesis testing, the test statistic (e.g. *F*, *t*, *r*) with confidence intervals, effect sizes, degrees of freedom and *P* value noted<br>*Give P values as exact values whenever suitable.*
☒	☐	For Bayesian analysis, information on the choice of priors and Markov chain Monte Carlo settings
☒	☐	For hierarchical and complex designs, identification of the appropriate level for tests and full reporting of outcomes
☒	☐	Estimates of effect sizes (e.g. Cohen's *d*, Pearson's *r*), indicating how they were calculated

Our web collection on statistics for biologists contains articles on many of the points above.

Software and code

Policy information about availability of computer code

Data collection	CellVoyager CQ1 and CQ1 Software Version 1.07.01.01 (Yokogawa Electric Corporation) was used for cell imaging. GloMax Navigator Microplate Luminometer and GloMax Navigator 3.0 (Promega) was used to measure luminescence. CytoFLEX Flow Cytometer S and CytExpert 2.4 (Beckman Coulter) was used to measure cellular fluorescence.
Data analysis	Luciferase assay data and flow cytometry data were analyzed in R (version 4.2.2) and Microsoft Office Excel (version 16.78). Fluorescent images were analyzed using ImageJ (version 1.52q). Flowjo (version 10.5.3) was used to visualize flow cytometry data. PyMOL (version 2.41) was used to visualize protein structures.

For manuscripts utilizing custom algorithms or software that are central to the research but not yet described in published literature, software must be made available to editors and reviewers. We strongly encourage code deposition in a community repository (e.g. GitHub). See the Nature Portfolio guidelines for submitting code & software for further information.

Data

Policy information about availability of data

All manuscripts must include a data availability statement. This statement should provide the following information, where applicable:

- Accession codes, unique identifiers, or web links for publicly available datasets
- A description of any restrictions on data availability
- For clinical datasets or third party data, please ensure that the statement adheres to our policy

All primary data can be accessed in the Source Data file. All other data including protein sequences and statistics are available within the article and Supplementary

information. Cited crystal structure data are available in the Protein Database (1HLL: https://doi.org/10.2210/pdb1HLL/pdb, 1P4B: https://doi.org/10.2210/pdb1P4B/pdb, 1QLN: https://doi.org/10.2210/pdb1QLN/pdb, 2Y0G: https://doi.org/10.2210/pdb2Y0G/pdb, 3ADF: https://doi.org/10.2210/pdb3ADF/pdb, 4PHY: https://doi.org/10.2210/pdb4PHY/pdb, 5A2Q: https://doi.org/10.2210/pdb5A2Q/pdb, 5VIV: https://doi.org/10.2210/pdb5VIV/pdb, 7BG1: https://www.rcsb.org/structure/7BG1) and the AlphaFold Protein Structure Database (AF-P0DMV8-F1: https://alphafold.ebi.ac.uk/entry/P0DMV8). Materials such as plasmids are available from the corresponding author upon reasonable request. No large datasets were generated, and no code was utilized in this manuscript.

Research involving human participants, their data, or biological material

Policy information about studies with human participants or human data. See also policy information about sex, gender (identity/presentation), and sexual orientation and race, ethnicity and racism.

Reporting on sex and gender	n/a
Reporting on race, ethnicity, or other socially relevant groupings	n/a
Population characteristics	n/a
Recruitment	n/a
Ethics oversight	n/a

Note that full information on the approval of the study protocol must also be provided in the manuscript.

Field-specific reporting

Please select the one below that is the best fit for your research. If you are not sure, read the appropriate sections before making your selection.

- [x] Life sciences
- [ ] Behavioural & social sciences
- [ ] Ecological, evolutionary & environmental sciences

For a reference copy of the document with all sections, see nature.com/documents/nr-reporting-summary-flat.pdf

Life sciences study design

All studies must disclose on these points even when the disclosure is negative.

Sample size	All the data include at least three biological replicates (n>=3). The sample sizes were determined based on general molecular and biological experiments and previously published literature (Kawasaki et al. 2023: https://doi.org/10.1038/s41467-023-37540-7 and Pu et al. 2017: https://doi.org/10.1021/acschembio.7b00532).
Data exclusions	Grubbs' test was used to detect and exclude the outlier in supplementary figure 4d. The outlier is included in raw data provided as Source data.
Replication	All experiments were performed in at least three biological replicates. There were no findings that could not be replicated or reproduced at least three times in independent experiments.
Randomization	Randomization was not used in this study because the same cell lines were grown under identical conditions and transferred to multiwell plates before transfection. All samples in the same experimental group were biological replicates.
Blinding	Blinding was not performed because the data were generated by using identical cells and automatic readout (flow cytometry, luminometer, and cell imager) and then analyzed through automated procedures (FlowJo, flowCore, Excel, and ImageJ).

Reporting for specific materials, systems and methods

We require information from authors about some types of materials, experimental systems and methods used in many studies. Here, indicate whether each material, system or method listed is relevant to your study. If you are not sure if a list item applies to your research, read the appropriate section before selecting a response.

Materials & experimental systems

n/a	Involved in the study
☐	☒ Antibodies
☐	☒ Eukaryotic cell lines
☒	☐ Palaeontology and archaeology
☒	☐ Animals and other organisms
☒	☐ Clinical data
☒	☐ Dual use research of concern
☒	☐ Plants

Methods

n/a	Involved in the study
☒	☐ ChIP-seq
☐	☒ Flow cytometry
☒	☐ MRI-based neuroimaging

Antibodies

Antibodies used	The commercial antibodies were not used in this study. We designed plasmid DNAs that encode the variable domains of the following antibodies fused with split RNAP fragments and transfected them into mammalian cells. For their information, see the Supplementary information and the links below.<br>Anti-GCN4 antibody (Hanes et al. 1998: https://doi.org/10.1073/pnas.95.24.14130 and Auf der Maur et al. 2002: https://doi.org/10.1074/jbc.M205264200)<br>Anti-FLAG antibody (Wegner et al. 2002: https://doi.org/10.1021/ac025922u)<br>Anti-EGFP antibody (Lim et al. 2018: https://doi.org/10.2144/000113964)<br>Anti-HCV IRES RNA antibody (Koirara et al. 2020: https://doi.org/10.1021/acschembio.9b00785)<br>Anti-Fluorescein antibody (Midelfort et al. 2004: https://doi.org/10.1016/j.jmb.2004.08.019)<br>Anti-Hsp70 antibody (Multhoff et al. 2007: https://doi.org/10.1016/j.ymeth.2007.06.006 and Friedrich et al. 2010: https://doi.org/10.1093/protein/gzp095)
Validation	The commercial antibodies were not used in this study. The variable domains of the antibodies were expressed in mammalian cells and evaluated in this study. The binding affinity and specificity of each antibody were previously evaluated in the following studies.<br>Anti-GCN4 antibody (Hanes et al. 1998: https://doi.org/10.1073/pnas.95.24.14130 and Auf der Maur et al. 2002: https://doi.org/10.1074/jbc.M205264200)<br>Anti-FLAG antibody (Wegner et al. 2002: https://doi.org/10.1021/ac025922u)<br>Anti-EGFP antibody (Lim et al. 2018: https://doi.org/10.2144/000113964)<br>Anti-HCV IRES RNA antibody (Koirara et al. 2020: https://doi.org/10.1021/acschembio.9b00785)<br>Anti-Fluorescein antibody (Midelfort et al. 2004: https://doi.org/10.1016/j.jmb.2004.08.019)<br>Anti-Hsp70 antibody (Friedrich et al. 2010: https://doi.org/10.1093/protein/gzp095)

Eukaryotic cell lines

Policy information about cell lines and Sex and Gender in Research

Cell line source(s)	Human embryonic kidney 293FT cell line (Thermo Fisher Scientific)
Authentication	The cell lines have been used for over three years in the lab. The cell lines were not additionally authenticated.
Mycoplasma contamination	Mycoplasma is regularly tested and the cell lines used in this study were negative for mycoplasma contamination.
Commonly misidentified lines (See ICLAC register)	No commonly misidentified cell lines were used.

Flow Cytometry

Plots

Confirm that:

☒ The axis labels state the marker and fluorochrome used (e.g. CD4-FITC).

☒ The axis scales are clearly visible. Include numbers along axes only for bottom left plot of group (a 'group' is an analysis of identical markers).

☒ All plots are contour plots with outliers or pseudocolor plots.

☒ A numerical value for number of cells or percentage (with statistics) is provided.

Methodology

Sample preparation	For flow cytometry analysis, cultured and transfected cells were washed once with PBS. Then, the cells were trypsinized and resuspended in culture medium. The resuspended cells were filtered through a mesh filter before flow cytometry analysis.
Instrument	CytoFLEX S Flow Cytometer (Beckman Coulter)

Software	CytExpert 2.4 (Beckman Coulter) was used to collect flow cytometry data. The flow cytometry data were analyzed using FlowJo software (version 10.5.3) and the "flowCore" package of R (vestion 2.10.0).
Cell population abundance	After excluding debris and doublets, 10000 or more single cells were collected and analyzed.
Gating strategy	The cells were first gated based on FSC-A/SSC-A to exclude the cell debris. Then, the cells were gated based on FSC-A/FSC-H to exclude the doublets and obtained single cells. The cell populations in the singlet gate were used for downstream analysis.

☒ Tick this box to confirm that a figure exemplifying the gating strategy is provided in the Supplementary Information.
